# Biomass Valorization Using Natural Deep Eutectic Solvents: What’s New in France?

**DOI:** 10.3390/molecules26216556

**Published:** 2021-10-29

**Authors:** Laura Wils, Soukaina Hilali, Leslie Boudesocque-Delaye

**Affiliations:** EA 7502 Synthèse et Isolement de Molécules BioActives (SIMBA), Université de Tours, 31 Avenue Monge, 37200 Tours, France; laura.wils@etu.univ-tours.fr (L.W.); soukaina.hilali@univ-tours.fr (S.H.)

**Keywords:** eco-valorization, biomass, Natural Deep Eutectic Solvent, extraction, France

## Abstract

With the growing interest in more environmentally friendly solvents and processes, the introduction of Natural Deep Eutectic Solvents (NaDES) as low cost, non-toxic and biodegradable solvents represent a new opportunity for green and sustainable chemistry. Thanks to their remarkable advantages, NaDES are now arousing growing interest in many fields of research such as food, health, cosmetics and biofuels. Around the world, NaDES are seen as a promising alternative to commonly used petrochemical solvents. The objective of this review is to draw up a panorama of the existing skills on NaDES in French laboratories and industries for the valuation of natural products. This review therefore focuses on current applications, skills and perspectives, in order to analyze the place of French research in the use of NaDES for the valorization of biomass since 2015.

## 1. Introduction

The actual transition to green and sustainable production processes led to intensive research of alternatives to solvent from petrochemicals or water [1]. In this context, Natural Deep Eutectic Solvents (NaDES) have been focusing the interest of researchers since the end of the 2010s. Numerous papers were published in various areas, such as analytical chemistry [2], biotechnology [3], energy [4], water treatment [5], food [6], cosmetic and pharmaceutics [7,8] or nanotechnology [9]. NaDES were first introduced by Choi et al. in 2013 [10]. They were defined as a mixture of hydrogen bond acceptor (HBA) and hydrogen bond donor (HBD) that could be found in natural resources such as sugars, amino-acids, fatty acids or organic acids [10,11,12]. Their association forms a network of hydrogen bonds that led to the lowering of the solid-mixture melting point [10,12]. NaDES are green solvents which are cost effective and simply prepared, and that are outcompeting existing organic solvents [13]. In addition they are renewable, biodegradable [12] and easily tunable [14]. Their unique solvent properties, including high extraction ability for some natural products and high solubilization strength of a wide range of organic and inorganic compounds, made them promising green media [10,11,12,13,14]. NaDES exhibited a wide range of polarity depending on their composition, from highly polar Choline chloride:Glycerol to non-polar C8:C12 [10,14]. NaDES reported in the literature were mainly hydrophilic with Choline Chloride or Betaine as the most recurring HBA combined with various HBD (sugars, polyols, amino acids). Recently, hydrophobic NaDES have emerged in the literature [15,16,17], that were composed of a combination of terpene (menthol, thymol) with sugars, polyols, or fatty acids or a combination of short-chain fatty acids [15,16,17].

**Figure 1:**
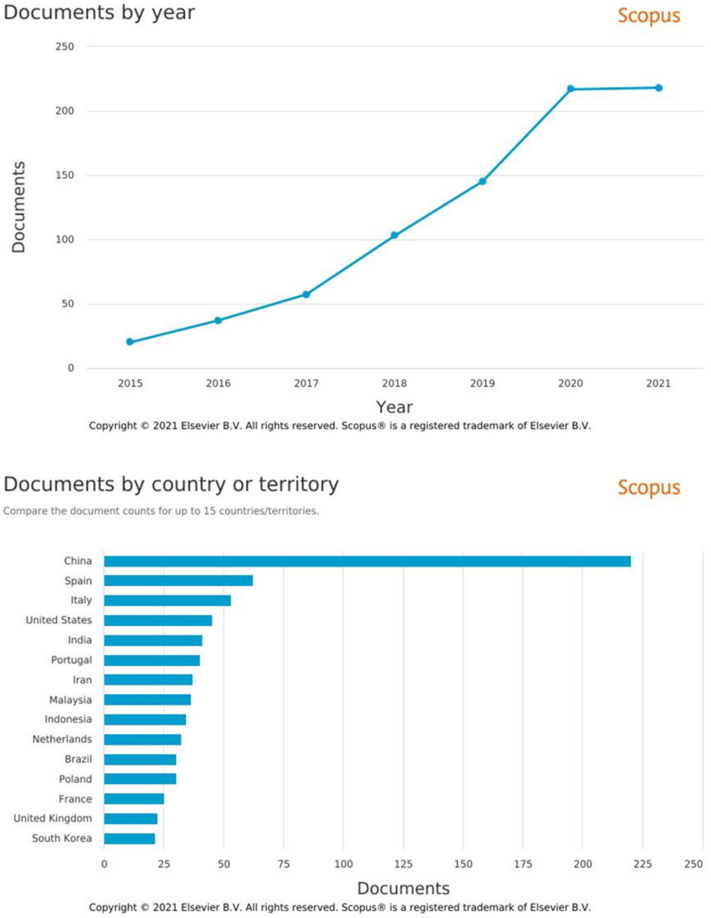
Bibliographic metrics from Scopus (https://www.scopus.com/, accessed on 28 September 2021), with “Natural Deep Eutectic Solvents” keywords, from 2015 to 2021.

NaDES is a highly competitive research field, as evidenced by the continuous increase in scientific production dealing with these issues since 2015 (Figure 1). China unsurprisingly occupies the first place in terms of number of publications (220), but the 10 best European countries combined have a higher publication rate (318), far ahead of the USA (Figure 1).

Focusing on European countries, France ranks 13th, far behind Spain in 2nd or Italy in 3rd, but in terms of valorization of natural biomass, it drops to 10th place.

France has intensively invested in biomass valorization with two main framework laws: the Law of Energy Transition and Green Growth (LTECV) (2015) and the law called “energy-climate”, promulgated in 2019. In fact, France represents a great resource of various biomasses such as marine biomass, agricultural residues, wood waste and forest residues [18]. The biomass cost is highly versatile thanks to climate change and biomass type, from 10-50 dollars per ton for wood waste to 10–50 dollars per kilogram for microalgae [18,19].

Although the production of energy or biofuel is currently the main outlet in France for biomass, the valorization of molecules with high added value is a rapidly expanding field, in particular with the rise of issues related to naturalness. The NaDES represent in this context a real opportunity for France to position itself as a leader in the industrial green transition. In this review we propose to analyze the French panorama of NaDES use for biomass valorization, considering each crucial step of a classical biorefinery scheme, before highlighting French industrial achievements in the field, in order to analyze the strengths and weaknesses of French research in this field.

## 2. Methodology

Publications and patents were included in the study according to the criteria listed below using Scopus as a database, in July 2021. Natural Deep Eutectic Solvents and Deep Eutectic Solvents were used as keywords to be found in the title or abstract of publications. The results were then refined according to the author’s country of affiliation (France), and reviews were excluded to focus on research articles and patents.

All the responses generated were then examined in order to exclude the references that did not deal with the subject of natural DES or the recovery of biomass.

**Figure 2:**
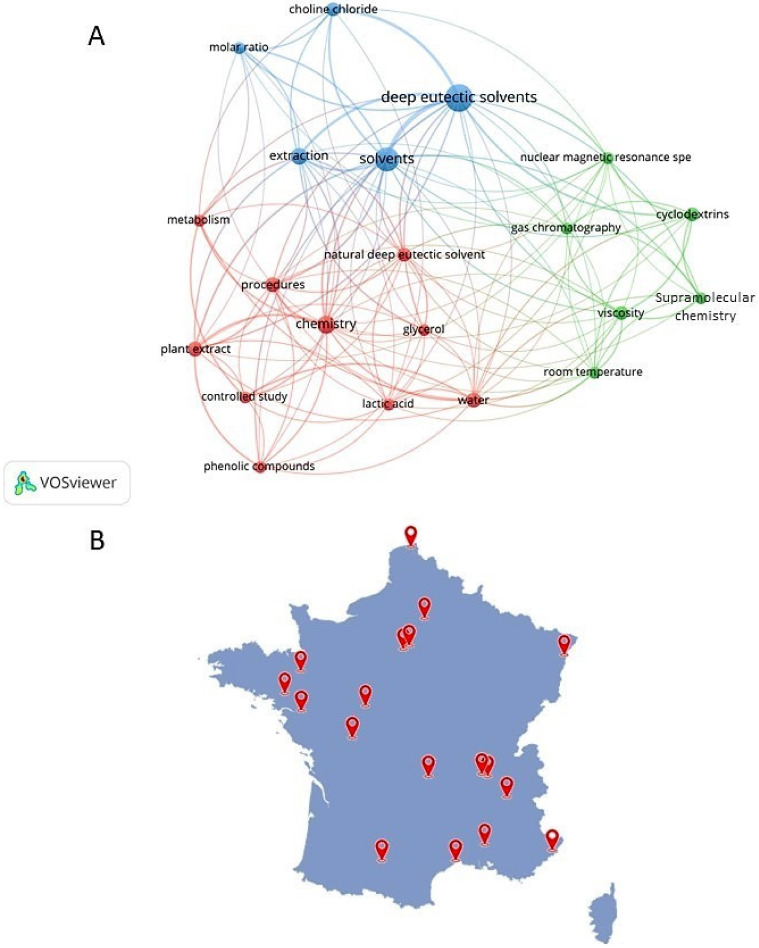
Concept map of bibliographic data of NaDES for biomass valorization in France (2015–2022), VoSViewer: co-occurrence of keywords appearing at least 3 times (**A**); Map of French laboratory location identified in the bibliographic set (**B**).

Nearly 250 references were then selected using the DES keyword and the French affiliation, brought down to 29 dealing with the recovery of biomass. Looking at the concept map generated by the analysis of this set of publications (Figure 2), three main groups were highlighted.

One cluster focused on the use of NaDES for plant extraction (red), with lactic acid, phenolic compounds and glycerol as the main occurrence. The second cluster is focused on DES and choline chloride dedicated to general extraction (blue), while the last cluster focused on the characterization of NaDES (green). We can also notice (Figure 2B) that the laboratories working on the NaDES are spread over the whole of France and not concentrated in a restricted geographical area.

**Figure 3:**
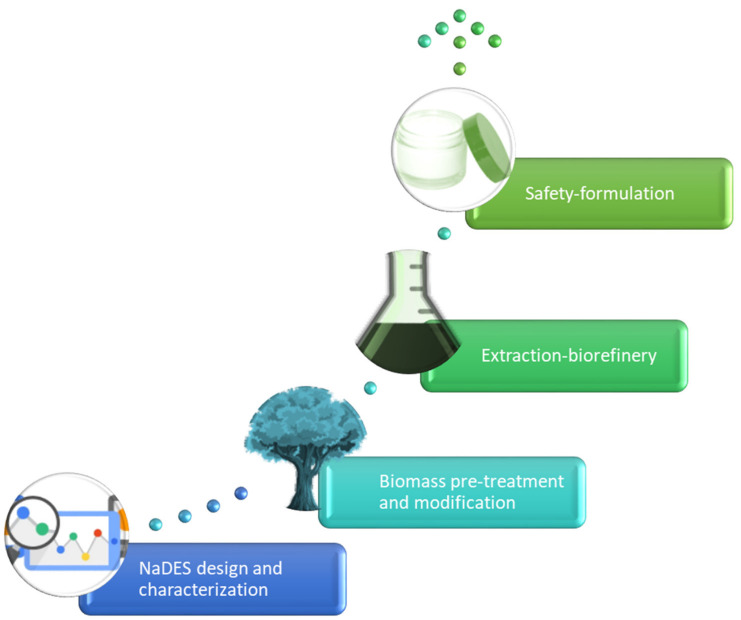
Workflow using NaDES for biomass-valorization.

In order to valorize the biomass, a classic multi-step workflow is generally used, summarized in Figure 3: (1) NaDES design / characterization; (2) pretreatment and modification of the biomass; (3) extraction and; (4) post-extraction steps such as toxicological studies and formulation.

The review will present the recent French achievement for each step, starting from NaDES design to toxicological assays.

## 3. NaDES Design and Physicochemical Characterization

### 3.1. Computational Theoretical Tools

By considering the wide range of raw materials that can enter into the composition of NaDES as well as the variety of possible combinations, theoretical computer models represent a promising tool to select, in silico, the most relevant NaDES more quickly. Their use would thus make it possible to reduce the time and resources required during the preliminary screening of NaDES in the laboratory.

#### 3.1.1. COSMO-RS

One of the widely used computational theoretical models is the COnductor-like Screening MOdel for Real Solvents (COSMO-RS). This model is usually used for the computation of the chemical potentials of targeted molecules in different solutions, thus allowing the estimation of numerous thermodynamic parameters, including the solubilities. This is a recognized theoretical approach to study the interactions between organic molecules and deep eutectic solvents. In a primary study by Milani et al., COSMO-RS was used for the prediction of the solubility of target solutes in 26 different DES in order to choose the right solvent for the extraction of steviol glycosides [20].

In another study by Chagnoleau et al. [21] six biphasic systems were investigated, as a way to optimize the purification and extractive diversity of five bio-compounds: quercetin, apigenin, coumarin, *β*-ionone, and *α*-tocopherol. The systems investigated biphasic were heptane-ethanol-(choline chloride/glycerol (1:2, mol/mol)), heptane-ethanol-(choline chloride/ethylene glycol (1:2, mol/mol)), heptane-ethanol-(choline chloride/levulinic acid (1:2, mol/mol)), heptane-ethanol glycerol, heptane-ethanol ethylene glycol, and heptane-ethanol-levulinic acid. The calculated and experimental outcomes of this study were in good agreement; thus, proving that COSMO-RS is a very useful and satisfactory tool for screening even complex biphasic solvents in order to obtain suitable systems for extraction and purification of distinct natural compounds

Again, the great diversity of existing NaDES makes it difficult to find the right system for specific processes, highlighting the need for in-silico tools such as COSMO-RS. This approach has thus demonstrated its practicality and its promising screening capacity to identify the appropriate system for the extraction and purification of the targeted bio-compounds. However, to perform the COSMO-RS calculation correctly, a prior geometric optimization using the TURBOMOLE software is essential in order to be able to consider the NaDES as an individual entity [20,21].

#### 3.1.2. Hansen Solubility Parameters

Hansen’s solubility parameters are a theoretical method used to predict the solubilization of target molecules in different solvents, in order to reduce the number of experiments required for the selection of the appropriate solvent for solubilization and extraction of target molecules from different biomass. It was used by El Kantar et al. [22] as a preliminary tool for the selection of the most suitable NaDES for the extraction of flavonoids, in particular naringin from grapefruit peels.

### 3.2. NaDES Physicochemical Properties Study

One of the most interesting characteristics of NaDES is their versatile physicochemical properties which can be adjusted for explicit requirements, thus opening up many possibilities for targeted extraction techniques [2].

Overall, many researchers have been interested in the characterization of the physicochemical properties of NaDES. Of all the properties, seven are considered the most critical from a process point of view: viscosity; phase diagram; density; the acid-base character; freezing point; polarity; and surface tension [23,24].

#### 3.2.1. Phase Diagram

Since NaDES were made of a mixture of two or more compounds existing in different physico-chemical states (solid-solid, solid-liquid, or liquid-liquid), the phase diagram of the NaDES system was important to explore. Classical approaches to determine the eutectic point usually involved calorimetric measurement, especially Differential Scanning Calorimetry.

Guinet et al. [25] studied the equilibrium phase diagram of a NaDES composed of citric acid and xylitol as well as the H-bond-type interactions forming between the components. Co-grinding of raw materials was used for the preparation of mixtures for microcalorimetric analysis. Indeed, the process can lead to the formation of mainly supra-molecular co-crystals with citric acid. Differential scanning calorimetry plots for mixtures are characteristic of what is typically found for eutectic mixtures and the formation of NaDES xylitol-citric acid is characterized by a sharp endothermic melting peak.

#### 3.2.2. Mass Spectrometry

Classical mass spectroscopy needed, in general, extensive NaDES dilution to avoid spectrometer probe degradation and to detect metabolites. It also involved the NaDES removal step, using, for example, solid phase extraction to preserve mass spectrometers. Recently, some groups have reported the use of mass spectroscopy to characterize NaDES itself.

Percevault et al. described an innovative Cold-Spray Ionization mass spectrometry (CSI MS) to investigate DES molecular organization. CSI MS is comparable to an Electrospray Ionization coupled with a nebulizing gas that is cooled using liquid nitrogen; it could be used as a tool to investigate the formation and stability of the structure of the supramolecular associations. CSI MS used it to analyze reline, i.e., Choline chloride: urea (1:2, mol/mol). [26] The ions detected on the CSI MS were distinctive of Choline Chloride salt and Urea association into a chloride adduct. It was found that an increase in the number of urea molecules does not imply a stabilization of the NaDES. In fact, it was suggested that the addition of urea in adducts of chlorides with cholinium cations would not result in the most stable ions. This study highlighted the potential of this mass spectrometry approach to the investigated NaDES molecular network in combination with other tools such as DSC or COSMO-RS.

#### 3.2.3. Water Quantification: Karl Fischer (KF) Titration and Vibrational Spectroscopy

Controlling the water content is a crucial point to consider when dealing with NaDES as it can have an impact on various parameters. It should be noted that a large number of NaDES consist of hygroscopic material, in particular choline chloride, which means that, during storage for example, the water content may increase in the NaDES or the extract. As discussed previously, NaDES could be adapted by adding water. Thus, to ensure the repeatability and reproducibility of the experiment, the water content should be quantified before it is used for the specific application, especially after a long storage period. The literature has different protocols and processes for quantifying water content, mainly gravimetric quantification, bound water fraction, Karl Fischer titration (KF) and Fourier transform infrared (FTIR). This latest powerful and reliable technique is widely used in many fields such as the food industry, analytical chemistry and pharmacy. The work carried out by Elderderi et al. study total infrared reflectance spectroscopy coupled with multivariate analysis for the quantification of water NaDES [27]. Three NaDES systems were studied including Betaine: Glycerol (1:8, mol / mol), Choline Chloride: Glycerol (1:2, mol / mol) and Glucose: Glycerol (1:3, mol / mol) with a water concentration range that varies from 0% to 40%. Although this work demonstrated the importance and efficiency of infrared analysis, water quantification was performed by depositing small volumes of NaDES on the infrared crystal, which requires a cleaning step between samples. Thus, in a complementary work carried out by the same team, the Raman technique was compared to the quantification of FTIR water [28]. Nowadays, Raman spectrum can be collected in situ directly from the sample container. While both techniques have had satisfactory results, Raman provides greater precision in estimating the concentration of samples. Both studies highlighted the relevance of these analytical tools to supporting the green solvent chemistry trend and development, including green and low-tech approach.

#### 3.2.4. Impact of NaDES Physical Properties on Biomass Valorization

Water could dramatically interfere with NaDES extraction performances, and could also be intentionally added in order to refine NaDES properties and thus meet the requirements of the desired application. In many cases, water improves the extractive performance of NaDES, especially when polyphenolic compounds are the target metabolites. However, adding water is controversial in the scientific community. Indeed, the presence of water not only affects the physicochemical properties of the solvent but can also compromise the integrity of the system [29].

Indeed, the increase in the water content considerably decreases the viscosity of NaDES and the latter depends closely on the strength of the bonds of the system. Stronger hydrogen bonds make the solvent more viscous, with a lower solubilization capacity. The addition of water is therefore sometimes necessary to facilitate their applications. However, if the water exceeds 50% by volume (sometimes 40%), a complete breakdown of the hydrogen bonds network could happen, leading to the total destructuring of the NaDES [29]. Below this ratio, water is an integral part of NaDES, which acts as the third compound and controls the system.

Moreover, the density of eutectic solvents has shown a temperature-dependent character. It seems that the density decreases linearly with increasing temperature [30]. Obviously, at high temperatures, molecules move faster and create more space. As a result, the density value decreases. Another hypothesis would be that the density of NaDES is linked to molecular packing and to the interactions between its constituents. The more ordered the system (i.e., rich in Van der Waals interactions and in hydrogen bonds), the more dense and viscous the system would be [15]. This is particularly the case for hydrophilic systems in which water has been added. According to Ribeiro et al., adding water tends to increase density by creating more hydrogen bonds until it reaches a breaking point when more than 50% water is added [31].

The density of the systems is therefore a function of the composition of the NaDES, its water content and the temperature of use.

The density will directly impact the viscosity of NaDES, which is a key parameter for transfer operations and extractive capacities [7,32]. Thus, several strategies have been described for lowering the viscosity of NaDES in order to improve their extractive performance and facilitate their use, in particular at the industrial level. The addition of a small proportion of water (less than 20%) is a strategy conventionally described for reducing viscosity and improving performance [15,29]. The second most common strategy consists in carrying out all the extraction and transfer operations while maintaining a high temperature, generally between 50 and 70 °C [7,32].

## 4. Biomass Pre-Treatment and Modification

Biomass is a broad category that includes all organic materials, vegetal or animal. In more general terms, the biological material defined here as biomass could take multiple forms: entire organism, organ, co-product, by-product, etc.

Pre-treatment is usually used to enhance the solid-liquid extraction yield and kinetics. The use of NaDES for such an end-goal has been discussed and investigated in literature; one of the most relevant works was Chen et al. [33]. The study focuses on the pre-treatment of apple pomace by exploring the impact of water-soluble pectin using 3 different NaDES. Thus, an important amount of pectin is still present in the remaining pomace after the one-step NaDES pre-treatment. Therefore, a complementary study was conducted by the same team [34], for a “greener” biorefinery scheme, where three NaDES systems (acidic, neutral, and alkaline); choline chloride:lactic acid, choline chloride:glycerol, and potassium carbonate:glycerol were investigated for a sequential NaDES pre-treatment method. Results presented in both studies open the way for a more novel pectin extraction via sequential pre-treatment based on recyclable NaDES, which can be integrated in a sustainable biorefinery process. In order to recover and recycle the different NaDES used in these pre-treatment strategies, precipitation using ethanol was performed, followed by a washing step with acetone. As NaDES were highly hydrophilic they remained in solution and were recovered after centrifugation and concentration under vacuum. For the non-conventional pre-treatment techniques, high voltage electrical discharges were studied by El Kantar et al. [22]. It is a process based on the electrical breakdown in the water used to improve biomolecules extraction. The study revealed that NaDES had potential as alternative solvents to enhance the polyphenol extraction from citrus fruits notably grapefruit peels in comparison to water. In this case NaDES remained in the final product and was not recovered.

Chitin is often extracted from insects as an alternative resource; still, the deacetylation of chitin into chitosan is considered an expensive procedure. Hence, Huet et al. compared the impact of ionic liquids (ILs) and DES on chitin pre-treatment on the efficiency of the deacetylation to obtain high quality chitosan from arthropod sources (*Bombyx eri* and *Hermetia illucens* and shrimp shells) [35]. It was found that chitosans with DES had more acetylation degrees than ILs. Overall, ILs are more suited for chitin amorphization and DES for preservation of α-chitin polymorphism and crystallinity; thus, offering covering numerous chitin’s applications. In this work, IL and NaDES were removed by extensive water wash.

## 5. Extraction

In the context of green chemistry, eco-extraction adopts these principles by targeting ever more natural extracts, to enhance both primary and secondary metabolites. Typically, the extraction of natural compounds is carried out through a process containing several unit operations, including pre- and post-processing with solid-liquid extraction as a key unit of operation. If it is not optimized, this step consumes time, energy and solvents (often obtained from petrochemicals) with a significant production of waste. In addition, depending on extraction conditions, the extract may be contaminated, denatured or contain a residual solvent, reducing its safety. Thus, eco-extraction is defined as the design and use of processes with reduced energy consumption, using alternative and renewable solvents, while ensuring a safe and high-quality extract [36]. The use of NaDES in place of a conventional solvent has led to an increase in unit operations, due to the additional step of preparing the solvent. This drawback is generally circumvented by eliminating the step of eliminating the NaDES, these solvents being recognized as biocompatible and safe [7,17,33,36].

### 5.1. Biomass Type

Extraction of natural compounds by NaDES has been reviewed by a great number of authors, thus illustrating the wide application areas of these new solvents. Nowadays, the French panorama covers numerous types of biomass ranging from eukaryotic to prokaryotic: plants, microalgae, fungi, yeasts and bacteria.

#### 5.1.1. Higher Plant

Several French teams have used higher plants as study models for eco-extraction. The GREEN laboratory (University of Avignon-INRAe, Avignon, France) has adopted *Stevia rebaudiana*, an aromatic plant from the *Asteraceae* family, well-known for the huge variety of glycosides present in its leaves. Among them, stevioside and rebaudioside A were the most abundant; they were responsible for the stevia sweet taste and having sweetening power about 250 and 450 times higher than saccharose, respectively [18]. Whereas, Gattefossé has selected *Calendula officinalis* flowers and more recently horse chestnut (*Aesculus hippocastanum*) to formulate flavonoid-enriched extracts [33].

#### 5.1.2. Microorganism

Numerous studies have also investigated the use of NaDES in biotechnology on lipid extraction from microorganisms. SIMBA laboratory (University of Tours, Tours, France) has described the first application of NaDES to non-pretreated microalgae for lipid and pigments extraction from the Cyanobacteria *Arthrospira platensis* (Spirulina) [17]. Whereas the CIRAD-Montpellier had contributed to a study orientated toward lipid extraction from the oleaginous yeast *Saitozyma podzolica* [37].

#### 5.1.3. By-Product

French teams are particularly devoted to promoting agro-waste. By-products are abundantly generated from food-processing industries and it is becoming a global concern. In this part we have illustrated some examples of international partnership between French and foreign universities.

A collaboration between the University of Rio de Janeiro and CIRAD (Montpellier, France) investigated the recovery of five typical phenolic acids from red and black rice bran using food-grade NaDES [38]. Rice bran corresponds to the outer shell of grain rice. It represents the main by-product generated during rice processing with around 29 million tons of global waste annually.

A biorefinery approach was engaged by Ruesgas-Ramón et al., to isolate phenolic acids from coffee and cocoa by-products. [39] Coffee and cocoa consumption generates solid wastes, such as pulp and husk, which represents million tons of waste. Coffee pulp represents around 40% of the fresh weight of coffee fruits, whereas cocoa husk and cocoa pod husk are the main co-products from the cocoa manufacturing. All these by-products contain high amounts of valuable biomolecules, such as phenolic compounds, fibers and alkaloids. Among them, the phenolic compounds represent the largest group of phytochemicals health benefits (anti-inflammatory, antiviral or antibacterial properties).

Every fruit consists of 15–50% of peel, seed or pulp, which are discarded as waste after juice extraction or fleshy part valorization. In the case of grapefruit, the volume of waste obtained is equivalent to the volume of the valorized product itself. Peels remain as the primary by-product, and are considered as a rich natural antioxidants ressources, according to their high level of polyphenols. Among the phenolic compounds found in grapefruit peels, naringin is the most abundant flavonoid, used as a food additive. Naringin extraction from grapefruit peels was successfully performed by El Kantar et al. (Lebanon-Compiègne partnership), using NaDES or aqueous glycerol [22].

Considering food waste, livestock and the consumption of meat also generate by-products that could be valorizated. For example, the poultry industry generates a vast amount of waste feathers annually, approaching 12 million tons of feathers in 2018 [40]. These feathers are usually discarded, incinerated or recycled as a low-value co-product. However, feathers contain approximately 90% of keratin, a fibrous protein found in hair. As a sustainable and biodegradable biopolymer, keratin has main possible applications. The processing of waste feathers using aqueous, inexpensive and food-grade DES was successfully demonstrated by Nuutinen et al., 2019 [40]; it was found that the DES NaOAc:Urea at the molar ratio (1:3) was able to dissolve 80% of the keratin by mixing the solution at 90 °C during 6 h.

Another team worked on cotton fibers obtained from the paper industry to extract cellulosic material [41].

### 5.2. Extraction Technique

#### 5.2.1. Ultrasound Assisted NaDES Extraction (UAE)

In the case of France, ultrasound is one of the most widely used methods and it can be illustrated by several studies. Undeniably, multiple studies published in recent years have proven ultrasound-assisted NaDES effectiveness for natural products extraction.

Ultrasonic waves accelerate the extraction process through the cavitation phenomena that generate bubbles inducing fragmentation and destabilization of the biological matrix, thus improving the mass transfer process. However, several parameters can highly affect the ultrasound-assisted extraction (UAE) process such as NaDES composition, sonication amplitude, extraction time, temperature, and water content. Their importance will be stated in the following section.

Wils et al. has conducted a screening of eighteen NaDES for the valorization of free fatty acids (FFA) and pigments from non-pretreated spirulina via ultrasound-bath assisted extraction [17]. They demonstrated that hydrophobic NaDES based on menthol or short chain fatty acids improved the extraction capacity of free fatty acids compared to organic solvents, ethyl acetate and dimethyl carbonate. Their FFA profile was predominated by saturated one (70%). The addition of water in sugar-based NaDES surprisingly enhanced the FFA recovery, especially of poly-unsaturated FFA.

The study carried out by Ruesgas-Ramón et al. [39] focused on the investigation of six NaDES based on Choline Chloride or Betaine as HBA for polyphenol extraction. Two different extraction conditions were compared: ultrasound-probe assisted extraction and heat stirring-assisted extraction. It was interesting to note that the type of assisted extraction method applied with NaDES can affect the extracted compounds (both quantitatively and qualitatively). Among the tested methods, UAE combined with choline chloride:lactic acid:water (1:2:1.5, mol/mol/mol) promotes the formation of a new compound, identified as furfural, in cocoa agro-waste that was not found in other conditions.

Milani et al. focused on the computational optimization of the extraction of glycosides from *Stevia rebaudiana* using UAE [20]. Sonication amplitude and temperature greatly influenced the efficiency of the method and were identified as key parameters. It was also shown that the amount of steviol glycosides through DES-UAE is almost three times higher than the one obtained by maceration.

Overall, UAE is widely recognized as an eco-friendly technique with reduced extraction time, an enhanced extraction efficiency, and a low solvent consumption. Ultrasound-assisted extraction coupled with NaDES now represent a green and sustainable alternative for the extraction of natural compounds.

#### 5.2.2. Microwave Assisted NaDES Extraction (MAE)

Microwave-assisted extraction (MAE) is an efficient, fast and effective extraction process that has increasingly been used as an alternative to conventional methods for natural products extraction. Microwaves damage cell walls by shattering under the sudden increase in temperature and internal pressure. This explosion mechanism explains the rapidity of MAE.

In France, Delavault et al. investigated the one-pot NaDES preparation/lipid extraction assisted by microwaves [37]. They shortened the extraction process through a single step approach, without using cell disruption pre-treatment. Eight NaDES essentially based on sugar, diol, choline chloride or betaine were investigated for microwave-assisted preparation. NaDES extract preparation with microwave heating was, in all cases, much faster than thermal heating, from a few seconds to a few minutes instead of thirty minutes for the conventional method. Additionally, it was possible to extract up to 70% fatty acid methyl esters from the non-pretreated yeast cells of *S. podzolica* with acidified choline chloride:xylitol with the one-pot process. Therefore, this new technique can save significant amounts of time and could reduce the energy input with highest efficiency.

#### 5.2.3. Miscellaneous

The search for green processes has led to the development of numerous processes, such as microextraction. The association of microextraction with deep eutectic solvents is quite new, with the first studies dating from 2014 [42].

Microextraction is a simple and low-cost process characterized by its high sensitivity, and very low solvent to solvent-free consumption [43]. It can be used for volatile and semi-volatile organic compounds determination from plant materials (e.g., alcohols, esters, aldehydes, hydrocarbons, ketones, terpenes, phenols, acids). Numerous microextraction processes were reported in the literature such as solid-phase microextraction, stir-bar sorptive extraction, hollow fiber liquid-phase microextraction, dispersive liquid-liquid microextraction, gas purge micro-syringe extraction, and headspace single drop microextraction. The latter was investigated in research conducted by Triaux et al. [44]. The aim of this study was to develop a new extraction method for terpenes from spices by coupling DES to headspace single-drop microextraction (DES-HS-SDME). DES-HS-SDME was performed by suspending a solvent drop at the tip of a microsyringe needle and exposing it to the headspace of a sample. The sample is heated to volatilize the target compounds. Then, the DES drop adsorbs the compounds volatilized from the sample matrix. After extraction, the suspended drop is retracted back into the microsyringe and transferred to a gas chromatography-mass spectrometer for further analysis. The choice of the extracting solvent is a crucial parameter. In this mode, the solvent should have adequate polarity, low volatility, low vapor pressure, and enough viscosity to ensure drop stability. Ten DES combinations were screened for the extraction of terpenes from nutmeg. The development of the method was optimized by the design of experiments. The DES N_4444_Br:dodecanol (1:2, mol/mol) showed the highest extraction efficiency and was selected to conduct optimization of the DES-HS-SDME method. The parameters tested are the extraction temperature, time, the drop volume and the sample mass. Only the two first parameters showed a significant influence on the efficiency of the method, indicating that (Na)DES are compatible with new green processes and can be a possible alternative to organic solvent for the extraction of bio-compounds.

Different NaDES used in biomass pre-treatment and extraction were summarized in Table 1.

## 6. Post-Extraction Step

### 6.1. Biological Evaluation

One of the drawbacks of DES was their non-volatility which can make their application very challenging, leading them to adapt biological test protocols to these new matrices.

Indeed, classical assay based on colorimetric measurement could be disturbed by the NaDES component leading to intensive research of alternative processes. For example, the direct electrochemistry of nine phenolic compounds was investigated for the first time in three DES-based media by cyclic voltammetry and differential pulse voltammetry in comparison with the Folin-Ciocalteu assay by Percevault et al. [26]. The Folin-Ciocalteu test is routinely employed for determining the total phenolic content in extracts; even though it is widely used in DES extracts [47], sometimes it can be difficult to handle or even inaccurate in some (Na)DES, while electrochemical techniques are quick, simple and do not require the use of metals or organic reagents. This study showed that electrochemical signals are well defined, even in a DES-medium, indicating that the redox activity of flavonoids is efficiently detected. Hence, electrochemical analyses can then constitute a potent alternative for the quantification of polyphenol contents. Polyphenols exhibited a better stability in the DES-water mixture, even after 10 months storage in a heat chamber at 30 °C. Moreover, the oxidation of quercetin was more difficult to achieve in DES-formulation than in aqueous conventional medium, suggesting strong interaction between DES components and the active substance. Antioxidant activity of flavonoids was also modulated through the choice of the DES. These results were very promising for pharmaceutical or cosmetic formulation.

In Caprin et al., antioxidant activity was assessed through the DPPH assay, measuring the radical scavenging activity [7,32]. NaDES-based sample preparation was not reported but NaDES did not seem to interfere into reaction between DPPH reagent and radical scavengers. NaDES extract showed a dose-related antioxidant activity with increasing concentrations of plant extract, significantly greater than the conventional solvent (ethanol/water, 50/50, *v/v*).

The use of samples in a eutectic medium also implied the use of a blank sample for each NaDES tested, these solvents possibly carrying a specific activity. In order to evaluate the impact of NaDES and Spirulina-NaDES-extracts on cutaneous inflammation, Wils et al. had infected Normal Human Epidermal Keratinocytes (NHEK) with *Staphylococcus aureus*, a bacterium known to induce skin inflammation [17]. The secreted levels of two inflammatory mediators (CXCL-8 and TNF-α) have been assessed by RT-qPCR and ELISA assay. Six NaDES and their resulting extract have been tested. The presence of NaDES or Spirulina-NaDES-extracts in the cell medium do not affect the release of pro-inflammatory mediators, except for extract formulated in Glucose:Glycerol:Water (1:2:4, mol/mol/mol) (GGW) which reduced the secreted level of these pro-inflammatory cytokines. This anti-inflammatory property of Spirulina-GGW was linked to high phycocyanin content, an antioxidant blue pigment produced by Cyanobacteria.

### 6.2. Toxicity Evaluation

Due to their natural origins (mostly primary metabolites), NaDES are generally considered as non-toxic solvents. However, “green” does not mean safe, and there is still controversy about their potential toxicity. However, there is still limited data on this topic (“NaDES and toxicity”, on Scopus shows only 74 articles).

One of the first in vivo studies was reported by Benlebna et al. [48]. The consequences of oral administration to rats of Betaine:Glycerol (1:2, mol/mol) extract enriched in polyphenols (from green coffee beans) was investigated. Lipid and glycogen contents as well as blood and liver oxidative stress were measured coupled with plasma and serum routine biochemical analyses. The polyphenol-enriched NaDES was delivered over a period of 14 days with a concentration of 6 mL/day per rat. Ten percent of water was added to the NaDES to decrease the viscosity and improve the administration. The rats that consumed NaDES extract showed a notable increase of plasma lipid levels, loss of appetite, increased water intake, plasma oxidative stress, and enlarged liver, kidney and stomach. Moreover, NaDES consumption induced mortality in two rats out of six. This work demonstrated some deleterious effects of oral administration of NaDES extract despite beneficial effects of polyphenols. The high NaDES oral dose or/and the NaDES composition were identified as the main causes, thus stating that both NaDES components and dose should be carefully selected and optimized for a safe formulation.

Cosmetic industry must now comply with European norms close to pharmaceutical ones. The French cosmetic company Gattefossé is a pioneer in cosmetic innovation using NaDES. Its research and development department recently published the good practices to follow in the purpose to use NaDES in cosmetics [7]. They studied especially the microbial safety profile and in vitro skill cell tolerance. Concerning microbial safety, microbial tests have to be achieved on NaDES to assess their efficacy of microbial conservation. The test consists of inoculating the sample with four strains: *Pseudomonas aeruginosa, Staphylococcus aureus, Candida albicans* and *Aspergillus brasiliensis*. It was performed on six sugar-based NaDES. The preservative properties of the sample are adequate if there is a significant decrease or no increase in the number of microbial colonies in the preparation after 2 days until 28 days. Fructose:Glycerol:Water (1:1:5, mol/mol/mol) surprisingly exhibited bactericidal activity on three strains out of four. NaDES toxicity was then evaluated using MTT assay on Normal Human Epidermal Keratinocytes (HNEK) and Normal Human Dermal Fibroblasts. Cells were grown in absence or in presence of 1% NaDES. No cytotoxicity was observed either on keratinocytes or fibroblasts after NaDES treatment (cell viability is above 80%). Consequently, those hydrophilic NaDES can be considered as harmless for a topical application.

For the same purpose, Wils et al. investigated the cytotoxicity of NaDES and their spirulina-extract (from 50 µg/mL to 200 µg/mL) on keratinocytes skin cells and four microbiota skin strains (*Staphylococcus aureus, Staphylococcus epidermidis, Cutibacterium acnes* and *Corynebacterium xerosis*) in vitro by XTT [17]. Six NaDES with different polarity were evaluated: three hydrophilic NaDES composed of glycerol, betaine or glucose, and three hydrophobic based on free fatty acid, menthol and organic acid. Unlike the previous study, sugar-based NaDES (and associated extracts) did not exhibit an antibacterial activity but favored the proliferation of commensal species. No cytotoxicity on HNEK was observed, confirming that some NaDES could be considered as harmless for skin cells. Non-polar NaDES and resulting extracts exhibited a dose-related toxicity towards staphylococci (90% at 200 µg/mL). In particular, *Staphylococcus aureus,* a pathogen strain linked to skin diseases, was impacted. This result opened the way to new approaches for NaDES use in skin microbiota regulation as well as antimicrobial preservations.

In vitro, cytotoxic activity of resveratrol (RES) formulated in NaDES and NaDES alone (from 0.5% to 2%) was assessed using the MTT assay on five different cell lines by Shamseddin et al. [49]. Malic Acid:ChCl (1:1, mol/mol) exhibited high toxicity at all tested concentrations on both human leukemia cells and primary human umbilical vein endothelial cells, whereas the cytotoxicity effect of 1,2-Propanediol:Choline Chloride:Water (1:1:1, mol/mol/mol) remained relatively moderated, even at the highest concentration (cell viability between 60 to 81%). This NaDES was then used to formulate RES (at 0.2 %). A dose-related toxicity was observed with increasing concentrations from 10 µM to 80 µM of RES-NaDES. The RES-NaDES and RES-DMSO exhibited similar toxicity when used at 40 and 80 µM, but RES dissolved in the NaDES remained soluble for a longer period.

### 6.3. Formulation

#### 6.3.1. Bio-Compounds Encapsulation

Due to their sensitivity to various physicochemical factors such as thermal or oxidation stress, bioactive compounds are often subjected to degradation. Encapsulation of bio-compounds was developed as key packaging technology to avoid degradation of sensitive metabolites. In this field, NaDES could bring an innovative perspective for more sustainable formulas. Basar et al. investigated β-carotene encapsulation in whey protein concentrate capsules through the technique of the emulsion electro-spraying using NaDES [45]. Electro-spraying is a method of liquid atomization using electrical forces, allowing dispersion into fine droplets without requiring high temperatures. Choline-based NaDES were screened in order to enhance the stability of *β*-carotene during the encapsulation process. *β*-carotene encapsulated using NaDES presented higher photo-oxidation stability compared with free β-carotene. This study unveiled the significant potential of ChCl-NaDES and electro-spraying for the encapsulation and stabilization of sensitive bio-actives with poor-water solubility.

#### 6.3.2. Volatiles Organic Compounds Encapsulation

Volatile organic compounds (VOC) are defined as one of the commonly known air pollutants released by commercial products (such as paint, cleaners…), chemical and petrochemical industries, but also natural products as essential oils. The volatile compound formulation is a central problem for essential oil, especially to extend their stability in end-product or to decrease their potential toxicity (irritation, phototoxicity). Di Pietro et al. discussed the possibility of a new hybrid material, called SUPRADES, that combined DES (ChCl:Urea) with *β*-cyclodextrin for a more effective encapsulation of VOC and especially essential oils [46]. These SUPRADES have demonstrated good performances for VOC absorption, similar to anethol, opening the way to a greener essential oils stabilization in food and cosmetics.

#### 6.3.3. Drug Formulation

In the pharmaceutical field, most of the active molecules were produced in a crystalline, poorly water-soluble form. Despite the drug stability in the crystalline state, its low bioavailability was a considerable obstacle to the development of new pharmaceuticals. Ionic liquids (ILs) have received considerable attention for their capabilities to stabilize pure solid chemicals in a liquid state. Unfortunately the use of ILs is limited in the pharmaceutical area due their potential toxicity and costs. NaDES are now explored as a greener alternative.

In Guinet et al., two poorly water-soluble active pharmaceutical ingredients (ibuprofen and acetaminophen) were dissolved in Citric Acid/Xylitol system and Raman spectroscopy was used for checking the amorphous character of the drugs and monitoring their stability in the formulation [25]. A great amount of crystalline state drugs (20 wt%) were dissolved in the NaDES, providing a strategy for stabilizing a great amount of amorphous drugs for pharmaceutical use.

Durand et al. studied the solubility of five antioxidants at 25 μM in 1,2-Propanediol:ChCl:Water (1:1:1, mol/mol/mol) [50]. Each antioxidant formulated in NaDES was evaluated for its Reactive Oxygen Species (ROS) scavenging effect, at short term (2 h), on fibroblast cells line using H_2_DCFDA reagent. Considering decyl rosmarinate and CR-6 (dimethylmethoxy chromanol), formulation in NaDES slightly enhanced ROS inhibiting action, respectively of 29% and 50%. It was interesting to note that the highest effects were observed with the antioxidants that exhibited the lowest ROS scavenging effect alone (i.e., CR-6 palmitate, Bis-Ethylhexyl Hydroxydimethoxy Benzylmalonate and Sinapine). Indeed, vectorization in NaDES led to a significant improvement of their antioxidant activity: more than 200%. Authors mentioned also that NaDES formulation may facilitate cellular membrane permeation.

Shamseddin et al. explored the feasibility of using NaDES to obtain new formulations of resveratrol, a plant polyphenol with a huge antioxidant potential [49]. Despite the well-known positive effects of resveratrol on human health, its low bioavailability and its poor water-solubility make it difficult to use in a therapeutic approach. Resveratrol formulation in NaDES (namely, 1,2-Propanediol:ChCl:Water (1:1:1, mol/mol/mol)) increased its solubilization and potentiated its MMP-9 inhibition ability [49]. This implies that some NADES could be possibly considered for a new generation of “drug formulation”.

Such NaDES-based formulations seem to increase bioavailability of drugs, probably through an improvement of cell membrane permeation capacity, or an optimization of activity within the intracellular medium. Durand et al. have conceptualized how NaDES may interact with membrane lipids or proteins [51]. The polar head of membrane lipids could bind to the NaDES and form a dynamic structure inside or around membranes. They postulated that NaDES could play a fundamental role in the transfer of substrates and/or products between enzymes, as well as the storage and transport of secondary metabolites [51].

#### 6.3.4. Enzyme Formulation

Bioconversion using enzymes has grown significantly in recent years, as part of the development of more eco-responsible processes. The highly selective character and the strong catalytic capacity of enzymes is combined with their renewability and biodegradability, as well as their capacity to work in mild operating conditions (ambient temperature, low pressure and neutral pH) [52]. The main bottleneck for the widespread use of enzymes is their low productivity and stability at high temperatures during the process. Several improvement strategies are currently under study [52]. The most common approaches used to improve enzyme stability are chemical modifications or immobilization on solid supports, however, these techniques often require unsustainable synthetic reagents. Delorme et al. investigated the laccase thermostability in aqueous NaDES-media as a new strategy [52]. Laccases are versatile enzymes which have recently found numerous applications as green catalysts in multiple fields such as the delignification of pulps in paper, fashion industry, or the removal of water-pollutants [53]. DES-systems were optimized in terms of ratio, composition and concentration to enhance laccase stability at high temperatures. Laccase activity was greatly improved when chlorine chloride was substituted by betaine, as the presence of chloride ions seemed to denature the enzyme. 

## 7. Industrial Development

In this section, only patents with French scientists or companies based in France have been considered without any time restriction. In the EspaceNet database, more than 1500 patents have already been published on eutectic solvents, mainly from China. In the French panorama, 13 patents were published between 2015 and July 2021.

Among these patents, 10 were dedicated solely to cosmetic use, 2 targeted the cosmetics, food and pharmaceutical market, and 1 concerned food and nutraceuticals (Table 2). It is interesting to note that only 4 companies were found as candidates: Yves Rocher (6 patents); Gattefossé (3 patents), Givaudan-Naturex (3 patents) and M&L Laboratories (1 patent). France being the world leader in cosmetic innovation, it is not surprising to find a large number of patents from French companies dealing with NaDES. Indeed, NaDES are part of the current challenges to the sector, which are naturalness and the reduction of water consumption. All the patents were focusing on hydrophilic NaDES, mainly composed of hexoses, organic acids, polyols and/or aminoacids, with possible water addition.

Two major types of patent were highlighted: general ones that protected a large [54,55] or reduced [60,63] number of NaDES for extraction or formulation; and specific patent describing the use of NaDES for one biomass or metabolites group usually referring to general patents previously described [54,55,56,57,58,59,61,63,64,66].

Among all of these, the latest patent of Givaudan-Naturex, with Scionix as a partner, is focusing on a new process to generate in situ NaDES during extraction by adding an exogenic amine to biomass [65].

Beside extraction, some patents also claimed a cosmetic use in formulation of final product or a benefit for skin as anti-ageing [53,54,66].

## 8. Strengths, Weaknesses, Opportunities and Threats (SWOTs) Analysis

**Figure 4:**
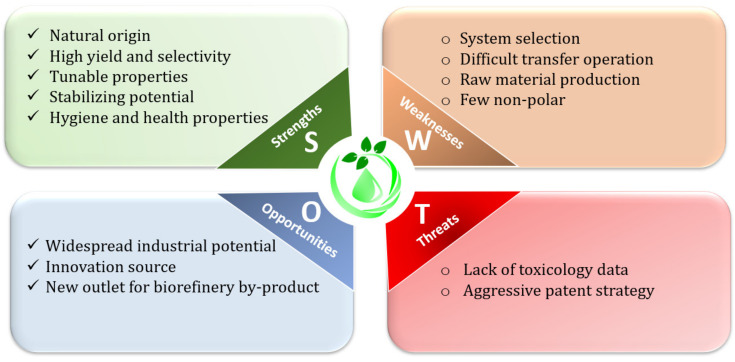
SWOT analysis of NaDES for biomass valorization.

SWOTs analysis is a strategic planning tool for examination, investigation, and identification of different factors and resources that may have negative or positive impact on the analyzed process. In this part, SWOTs analysis was used to study the different factors impacting the valorization of biomasses using NaDES favorably or unfavorably (Figure 4).

Strengths: NaDES are depicted as sustainable, low-cost and biodegradable solvents due to the natural origin of their constituents. The components used to form NaDES are primary metabolites that are commonly found in food, cosmetic or pharmaceutics. Numerous studies showed higher extraction performances (yield, selectivity) of NaDES compared to regular solvent, coupled with an enhanced stability of bio-compounds during both extraction and storage [14,66,67]. Additionally, NaDES are compatible with modern extraction processes (ultrasounds, microwaves) [36]. NaDES also have almost zero vapor pressure, meaning less air pollution.

Weaknesses: A major limit to the NaDES development and widespread use is the wide variety of existing systems and the low availability of predictive selection tools. A know-how is usually required to select a NaDES without extensive preliminary screening. In addition, few NaDES systems were dedicated to non-polar metabolites, despite the urgent need of greener alternatives to alkane. Another disadvantage is the density and viscosity of NaDES that could disturb transfer operation, thus limiting industrial application. Finally, raw materials of NaDES, if natural, were not always prepared via biorefinery or green processes and could also lead to competition with the food market.

Opportunities: As discussed in this review, biomass valorization by NaDES has opened the path towards numerous prospects, notably in the food, cosmetic and pharmaceutical industries. The unique physical properties of NaDES could lead to innovative processes or approaches to remove current technological barriers that limit their industrial spread. In addition, the increasing demand for NaDES raw material represents a new outlet for by-product that will boost the development of the biorefinery process.

Threats: The lack of data considering real NaDES toxicological impact is a major threat to their use, especially in the health industry. While NaDES are less toxic than conventional solvents, this does not prove that they will not present any negative effects to human health, especially concerning chronic use. A second important threat lies in the fact that most of NaDES combinations and applications are patented, and new patents are published every month.

## 9. Conclusions

The use of NaDES to valorize biomass is a dynamic field of research which has real industrial potential. France, even with a late start compared to Spain and Italy, is a major player in innovation in the field of NaDES, in particular for cosmetic applications.

This review highlighted the versatility of French researchers at all stages of biomass recovery with two major specificities: the recovery of by-products and innovative characterization tools.

The upcycling of agricultural waste has been intensively studied and the most representative extraction protocol involved the use of ultrasound.

In the post-extraction stages, safety investigations vis-à-vis the skin, the microbiota or the liver were the most dynamic areas, along with the formulation.

## Figures and Tables

**Table 1:** NaDES used in biomass pre-treatment, extraction and formulation.

	NaDES			Ratio	Used Processes	Matrix	Metabolite	Ref.
	Component 1	Component 2	Component 3					
Pre-treatement	Choline Chloride	lactic acid	-	1:2	stirring and heating	apple pomace	pectine	[33]
		urea	-	1:2				
		oxalic acid	-	1:2				
	Choline Chloride	lactic acid	-	1:2	NaDES extraction followed by EtOH and acetone wash	apple pomace	pectine	[32]
		Glycerol	-	1:2				
	Choline Chloride	lactic acid	-	1:3	high voltage electrical discharges	grapefruit peels	polyphenols (naringin)	[22]
		tartaric acid	-	1:3				
	Glucose	Lactic acid	-	1:5				
	Glycine	Lactic acid	-	1:3				
Extraction	Betaine	Glycerol	-	1:2, 1:4, 1:8	ultrasound-assisted extraction	spirulina	fatty acids and pigments	[17]
	Glucose	Glycerol	-	1:2, 1:3, 1:4, 1:5				
		Glycerol	Water	1:2:2, 1:2:4				
		Glycerol	Betaine	1:2:4				
	Lactic acid	Betaine	-	2:1				
		Glycerol	-	1:1				
	Menthol	Lactic acid	-	1:2				
		Levulinic acid	-	1:2				
		Octanoic acid	-	1:1				
	Octanoic acid	Lauric acid	-	3:1				
	Nonanoic acid	Lauric acid	-	3:1				
	Nonanoic acid	Decanoic acid	Lauric acid	3:2:1				
	Choline Chloride	Lactic acid	Water	1:2:1.5	ultrasound-assisted extractionstirring and heating extraction	coffee and cocoaby- and co-products	gallic acid, chlorogenic acid, caffeine, theobromine, furfural	[39]
		Glycerol		1:2:1.5				
		1,4-Butanediol		1:2:1.1				
	Betaine	Lactic acid		1:2:1.5				
		Glycerol		1:2:1				
		1,4-Butanediol		1:2:1				
	Choline Chloride	Arabinose	-	1:1	microwave-one pot assisted extraction	yeast *Saitozyma podzolica*	lipid extraction and glycolipid production	[37]
		Glucose	-	2:1				
		Urea	-	1:2				
		Glycerol	-	1:2				
		1,2-Propanediol	-	1:1				
		Saccharose	-	4:1				
		Xylitol	-	1:1				
		Sorbitol	-	1:1				
	Betaine	Glycerol	-	1:2				
		1,4-Butanediol	-	1:4				
	Choline Chloride	1.2-propanediol	Water	1:1:1	orbital agitation	rice co-products	phenolic compounds	[38]
		Lactic acid	-	1:10				
	Choline Chloride	Oxalic acid	-	1:1	glass reactor	cotton fiber	cellulose nanocrystals	[41]
Microextraction	Choline Chloride	Urea	-	1:2	single-drop microextraction	cinnamon, cumin, fennel, clove, thyme, and nutmeg	terpenes	[44]
		Lactic acid	-	2:3				
Encapsulation	Choline Chloride	propanediol	water	1:1:1	encapsulation by emulsion electrospraying in whey protein concentrate	-	*β*-carotene	[45]
		Glucose		5:2:5				
		Glycerol	-	1:2				
		butanediol	-	1:2				
	Choline Chloride	Urea	Cyclodextrin	1:2 (10%)		-	volatile compounds	[46]

**Table 2:** Patents with French applicants published since 2015.

Company	Patent	NaDES Raw Materials	Biomass/Metabolites	Market	Ref
Yves Rocher	FR 3017292-A1	Hexoses Organic acids Polyols Aminoacids	active cosmetic ingredient	Cosmetics	[54]
	FR 3046352-A				[55]
	WO 2019/081859-A1		*Phytelephas sp.*		[56]
	WO 2019/063929-A1		*Syringa vulgaris*		[57]
	WO 2019/063927-A1				[58]
	FR 3042415-A1		*Glycyrrhiza glabra*		[59]
Gattefossé	FR 3036618-A1	Sugar Polyols Aminoacids (Betain)	Plants	Cosmetics	[60]
	FR 3068352-A1		Stilbenoids		[61]
	FR 3067939-A1	Fructose/Glycerol/water	*Withania somnifera*		[62]
Givaudan-Naturex	FR 3034625-A1	Betain Polyols Organic acids	plant, animal, procaryote	Cosmetic Food Pharmaceutic	[63]
	FR 3049864-A1		*Aerva sp.*		[64]
	WO 2019/219774-A2	Exogenic amine	plant, animal, procaryote	Food Pharmaceutic	[65]
Laboratoires M&L	FR 3067604-A1	Organic acids Sugars Polyols Choline salts Aminoacids	*Helichrysum italicum*	Cosmetics	[66]
